# The Impact of Concomitant Empiric Cefepime on Patient Outcomes of Methicillin-Resistant *Staphylococcus aureus* Bloodstream Infections Treated With Vancomycin

**DOI:** 10.1093/ofid/ofz079

**Published:** 2019-04-03

**Authors:** Evan J Zasowski, Trang D Trinh, Safana M Atwan, Marina Merzlyakova, Abdalhamid M Langf, Sahil Bhatia, Michael J Rybak

**Affiliations:** 1 Anti-Infective Research Laboratory, Department of Pharmacy Practice, Eugene Applebaum College of Pharmacy and Health Sciences, Wayne State University, Detroit, Michigan; 2 Department of Pharmacy Practice and Translational Research, University of Houston College of Pharmacy, Houston, Texas; 3 Department of Clinical Sciences, Touro University California College of Pharmacy, Vallejo, California; 4 Department of Clinical Pharmacy, UCSF School of Pharmacy, San Francisco, California; 5 Division of Infectious Diseases, Department of Medicine, School of Medicine, Wayne State University, Detroit, Michigan; 6 Department of Pharmacy Services, Detroit Medical Center, Detroit, Michigan

**Keywords:** bacteremia, beta-lactam, combination therapy, MRSA, synergy

## Abstract

**Background:**

Data suggest that vancomycin + β-lactam combinations improve clearance of methicillin-resistant *Staphylococcus aureus* (MRSA) bloodstream infections (BSIs). However, it is unclear which specific β-lactams confer benefit. This analysis evaluates the impact of concomitant empiric cefepime on outcomes of MRSA BSIs treated with vancomycin.

**Methods:**

Retrospective cohort study of adults with MRSA BSI from 2006 to 2017. Vancomycin + cefepime therapy was defined as ≥24 hours of cefepime during the first 72 hours of vancomycin. The primary outcome was microbiologic failure, defined as BSI duration ≥7 days and/or 60-day recurrence. Multivariable logistic regression was used to evaluate the association between vancomycin + cefepime therapy and binary outcomes. Cause-specific and subdistribution hazard models were used to evaluate the association between vancomycin + cefepime and BSI clearance.

**Results:**

Three hundred fifty-eight patients were included, 129 vancomycin and 229 vancomycin + cefepime. Vancomycin + cefepime therapy was independently associated with reduced microbiologic failure (adjusted odds ratio [aOR], 0.488; 95% confidence interval [CI], 0.271–0.741). This was driven by a reduction in the incidence of BSI durations ≥7 days (vancomycin + cefepime aOR, 0.354; 95% CI, 0.202–0.621). Vancomycin + cefepime had no association with 30-day mortality (aOR, 0.952; 95% CI, 0.435–2.425). Vancomycin + cefepime was associated with faster BSI clearance in both cause-specific (HR, 1.408; 95% CI, 1.125–1.762) and subdistribution hazard models (HR, 1.264; 95% CI, 1.040–1.536).

**Conclusions:**

Concomitant empiric cefepime improved MRSA BSI clearance and may be useful as the β-lactam component of synergistic vancomycin + β-lactam regimens when empiric or directed gram-negative coverage is desired.

Methicillin-resistant *Staphylococcus aureus* (MRSA) is a serious public health threat resulting in thousands of infections and deaths annually [1]. A major contributor to the associated morbidity and mortality is MRSA bloodstream infection (BSI) [2, 3]. Vancomycin has been the treatment of choice for MRSA BSI for decades, but treatment failure rates are in excess of 30% [4–6]. Despite availability of newer alternative anti-MRSA treatment options, none have been shown to be conclusively superior to vancomycin for MRSA BSI [6–10]. Combination therapy, such as vancomycin plus an aminoglycoside, has also been used in attempt to optimize efficacy. However, this approach fell out of favor due to increased risk of nephrotoxicity, coupled with lack of data to demonstrate improved treatment outcome [11].

The concept of combination therapy for MRSA BSI is being reexamined, as researchers and clinicians have begun to further explore previously identified synergy between glycopeptide and β-lactam antibiotics [12]. Synergy between vancomycin and several β-lactams, including nafcillin, cefazolin, piperacillin/tazobactam, cefepime, and ceftaroline, has been well documented in vitro and in vivo [13–17]. This synergy appears to translate to the clinical setting. A pilot randomized controlled trial of vancomycin plus flucloxacillin vs vancomycin monotherapy demonstrated shortened BSI duration in the combination group [18]. Observational studies of vancomycin given in combination with β-lactams empirically in patients ultimately found to have MRSA BSI also suggest improved microbiologic clearance [19, 20].

Although a vancomycin + β-lactam approach for MRSA BSI shows promise, challenges remain before it can be translated into practice. The majority of the clinical evidence involves an amalgamation of different β-lactam antibiotics [19, 20]. This makes it difficult to discern which specific β-lactams provide clinical benefit and which are best suited for this purpose. This is particularly true in the United States, where flucloxacillin, the single agent with the strongest clinical evidence at the present time, is not commercially available [18]. Although ongoing studies may address this need by studying specific narrow-spectrum β-lactams, clinical data evaluating broad-spectrum β-lactams will still be needed [21]. Such data would be crucial in adopting a vancomycin + β-lactam approach to inform empiric prescribing before MRSA is identified and directed therapy when patients require continued gram-negative coverage. Given the frequency with which cefepime is used concomitantly with vancomycin for empiric antibiotic therapy for suspected antibiotic-resistant infections and the in vitro synergy between these 2 agents, the objective of this analysis was to evaluate the impact of concomitant empiric cefepime on patient outcomes of MRSA BSI treated with vancomycin.

## METHODS

### Study Design and Population

This was a retrospective, observational cohort study of adult patients with MRSA BSI from 2006 to 2017 at the Detroit Medical Center (DMC), an eight hospital healthcare system in Southeast Michigan. Patients aged ≥18 years with ≥1 positive blood culture for MRSA initially treated with ≥72 hours of vancomycin were eligible for inclusion [22]. Patients who received ≥24 hours of concomitant MRSA-active therapy or a β-lactam other than cefepime during the initial 72 hours of vancomycin therapy were excluded. Patients without follow-up blood cultures and those with repeat MRSA BSI episodes were also excluded. Patients who received ≥24 hours of concomitant cefepime during the initial 72 hours of vancomycin therapy were considered to have received vancomycin + cefepime. Patients who received <24 hours of cefepime were considered to have received vancomycin monotherapy. This study was approved by the DMC Research Review Committee and the institutional review board at Wayne State University (WSU), and a waiver of informed consent was granted.

### Patient Data Elements and Collection

Methicillin-resistant *Staphylococcus aureus* BSIs for inclusion were identified through a list of positive MRSA blood cultures at the DMC during the study period. Patient data were extracted from the medical record by trained reviewers using a structured data collection form within the Research Electronic Data Capture (REDCap; Vanderbilt University) data capture tool hosted at WSU [23]. Data elements included demographics, medical history, comorbid conditions, antibiotic therapy and associated laboratory parameters, infectious diseases consult, and pursuit of source control. The degree of patient comorbidity was quantified using the Charlson comorbidity index [24]. Severity of illness was quantified using the Acute Physiology and Chronic Health Evaluation II (APACHE II) score using the worst physiological parameters within 24 hours of index MRSA blood culture [25]. The source of the MRSA BSI was based on the treating physician’s notes and available clinical/diagnostic data. Microbiologic data including antibiotic susceptibilities by Microscan, Phoenix, and/or Etest were collected from the medical record.

### Outcomes

The primary outcome was microbiologic failure, defined as a BSI duration ≥7 days and/or MRSA BSI recurrence within 60 days of the end of MRSA BSI therapy. Secondary outcomes included each individual component of microbiologic failure, mortality from any cause within 30 days of index MRSA blood culture, time to MRSA BSI clearance in days, and hospital-length of stay (LOS) post–MRSA BSI onset in days.

### Data Analysis

The primary analysis focused on examining the association between concomitant vancomycin + cefepime therapy and microbiologic failure. Bivariate analysis was conducted comparing covariates between patients who did and did not experience microbiologic failure. Categorical variables were compared between groups using the χ^2^ or Fisher exact test, whereas continuous variables were compared using the Student *t* test or Mann Whitney *U* test. The independent association between concomitant vancomycin + cefepime therapy and microbiologic failure was then examined using multivariable logistic regression. Vancomycin + cefepime therapy, along with all the variables associated with microbiologic failure at a *P* value <.2 in bivariate analysis with biologic plausibility, were entered into logistic regression models simultaneously and removed in a backward, stepwise fashion. Covariates were retained in the final model if the *P* value for the likelihood ratio test for their removal was <.1. Because vancomycin + cefepime was the exposure of interest, it was forced to remain in the final step of the regression models even if no statistical association was observed. Model fit was assessed with the Hosmer-Lemeshow goodness-of-fit test; models with a nonsignificant result were considered adequate. Multicollinearity of candidate regression models was assessed via the variance inflation factor, with values between 1 and 5 considered acceptable.

Given the imbalance in the proportion of patients with an endovascular BSI source between treatment groups and the well-documented association between endovascular sources and prolonged, recurrent BSI, a secondary post hoc matched analysis was conducted to further examine the association between vancomycin + cefepime therapy and microbiologic failure. Patients were matched 1:1 on endovascular source, and conditional logistic regression was conducted on the resulting matched data set as described above for the primary analysis.

Additional secondary analyses were conducted to examine the association between vancomycin + cefepime therapy and the secondary outcomes. Any statistically significant associations between vancomycin + cefepime therapy and secondary outcomes in bivariate analysis were examined further through multivariable analysis using model-building strategies congruent with those explained above for the primary analysis. The independent association between vancomycin + cefepime therapy and BSI clearance was evaluated using both a cause-specific proportional hazard model (patients experiencing mortality before BSI clearance were censored) and a subdistribution hazards model (simultaneous model of survival and BSI duration) to account for the competing risks of death and BSI clearance [26].

All statistical tests were 2-sided; *P* values ≤.05 were considered statistically significant. Analyses were performed using SPSS Statistics, IBM SPSS software, version 25.0 (IBM Corp., Armonk, NY), and SAS, version 9.4 (Cary, NC).

## RESULTS

A total of 358 patients were included. A full description of demographics, clinical characteristics, and outcomes of the cohort is available in Supplementary Table 1. The cohort was predominantly African American (79.6%), majority male (64.8%), and had a median (interquartile range [IQR]) age of 60 (52–69) years. Common comorbidities were diabetes (38.0%), moderate/severe renal disease (35.5%), chronic hemodialysis (25.4%), heart failure (23.5%), intravenous drug use (15.1%), and liver disease (15.6%). The median (IQR) Charlson comorbidity index and APACHE II scores were 3 (1–5) and 18 (11–24), respectively. The most common MRSA BSI sources were endovascular (23.5%), lower respiratory tract (21.5%), skin and soft tissue (21.2%), and intravenous catheter (20.4%). The median (IQR) duration of vancomycin therapy was 5 (4–9) days. The majority of patients (79.5%) had their vancomycin therapy dosed and monitored to a trough concentration target range of 15–20 mg/L, whereas the remaining had their vancomycin therapy dosed and monitored to an AUC of 400–600 mg*h/L [27, 28]. Two hundred twenty-nine patients (64.0%) received concomitant cefepime therapy with a median (IQR) duration of 3 (2–4) days. The most common cefepime dose and interval were 1000 mg (52.4%) and every 8 hours (41.5%), respectively. Microbiologic failure was observed in 107 patients (29.9%), with 83 (23.3%) having a BSI duration ≥7 days. Thirty-day mortality was observed in 57 (15.9%) patients.

**Figure 1.**
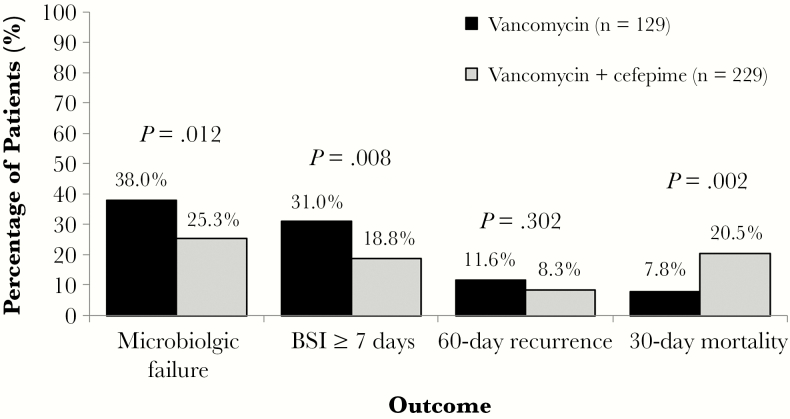
Comparison of efficacy outcomes between patients receiving vancomycin or vancomycin + cefepime. Abbreviation: BSI, bloodstream infection.

Bivariate comparisons between patients receiving vancomycin or vancomycin + cefepime are displayed in Table 1. Patients receiving vancomycin + cefepime were significantly older, had a higher proportion of baseline acute kidney injury, and had significantly higher APACHE II scores. Although select individual comorbid conditions differed between groups, no difference in Charlson comorbidity index was observed. Patients receiving vancomycin + cefepime were more likely to have been admitted from a nursing facility or have a polymicrobial bloodstream infection. Endovascular MRSA BSI source, including endocarditis, and lower respiratory tract source were more common among vancomycin + cefepime patients, whereas skin and soft tissue and bone/joint source were more common among vancomycin monotherapy patients. Patients in the vancomycin + cefepime group were more likely to have AUC-guided vancomycin therapy. Microbiologic failure was significantly more common among vancomycin monotherapy patients (Figure 1). This difference was primarily driven by a greater incidence of BSI durations ≥7 days in the vancomycin monotherapy group. No difference in the incidence of 60-day recurrence was observed. Thirty-day mortality was significantly more common among vancomycin + cefepime patients. No difference in safety outcomes, including vancomycin-associated nephrotoxicity, neurotoxicity, or CDI, was observed between treatment groups (Table 1).

**Table 1.** Bivariate Comparisons of Demographics, Clinical Characteristics, and Outcomes Between Patients Receiving Vancomycin or Vancomycin + Cefepime

Covariate	Vancomycin^a^ (n = 129)	Vancomycin + Cefepime^a^ (n = 229)	*P* Value
Demographics			
Age, y	56 (48–66.5)	61 (53.5–71)	.001
Male	86 (66.7)	146 (63.8)	.580
Race			.548
African American	105 (81.4)	180 (78.6)	
Caucasian	20 (15.5)	45 (19.7)	
Hispanic	2 (1.6)	3 (1.3)	
Other/unknown	2 (1.6)	1 (0.4)	
Comorbidities & medical history			
Myocardial infarction	4 (3.1)	20 (8.7)	.047
Heart failure	26 (20.2)	58 (25.3)	.268
Peripheral vascular disease	27 (20.9)	43 (18.8)	.622
Cerebrovascular disease	18 (14.0)	45 (19.7)	.174
Dementia	8 (6.2)	36 (15.7)	.008
Chronic pulmonary disease	25 (19.4)	66 (28.8)	.049
Chronic obstructive pulmonary disease	21 (16.3)	59 (25.8)	.039
Asthma	5 (3.9)	12 (5.2)	.560
Connective tissue disease	19 (14.7)	24 (10.5)	.235
Peptic ulcer disease	2 (1.6)	5 (2.2)	1.000
Liver disease	25 (19.4)	31 (13.5)	.144
Mild^b^	23 (17.8)	27 (11.8)	.114
Moderate/severe^c^	2 (1.6)	4 (1.7)	1.000
Diabetes	47 (36.6)	89 (38.9)	.649
With end-organ damage	37 (28.7)	56 (24.5)	.381
Hemiplegia	3 (2.3)	5 (2.2)	1.000
Moderate/severe renal disease^d^	41 (31.8)	86 (37.6)	.273
Chronic hemodialysis	29 (22.5)	62 (27.1)	.338
Solid tumor without metastasis	2 (1.6)	5 (2.2)	1.000
Leukemia	0	0	—
Lymphoma	0	0	—
Metastatic solid tumor	5 (3.9)	8 (3.5)	1.000
HIV	5 (3.9)	7 (3.1)	.679
AIDS	3 (2.3)	2 (0.9)	.261
Charlson comorbidity index	2 (1–5)	3 (1–5)	.303
Intravenous drug use	25 (19.4)	29 (12.7)	.088
Prior hospitalization (90 d)	48 (37.2)	97 (42.4)	.341
Prior IV vancomycin (90 d)	31 (24.0)	51 (22.3)	.704
Prior MRSA infection (1 y)	26 (20.2)	28 (12.2)	.044
Clinical data			
Admitted from:			.011
Home	98 (76.0)	146 (63.8)	.017
Nursing facility	20 (15.5)	68 (29.7)	.003
Transferred from another hospital	11 (8.5)	15 (6.6)	.489
Weight, kg	75 (66.8–87.5)	76.9 (65.1–91.6)	.541
Creatinine clearance,^e,f^ mL/min	72.9 (49.2–98.1)	56.5 (33.2–89.2)	.009
Acute kidney injury^f^	34 (26.4)	84 (36.7)	.046
APACHE II score^f^	13 (8–19)	20 (15–27)	<.001
Neutropenia^f^	0	1 (0.4)	1.000
Infection data			
Vancomycin MIC,^g^ mg/L			.834
2	50 (38.8)	96 (41.9)	
1	78 (60.5)	131 (57.2)	
≤0.5	1 (0.8)	2 (0.9)	
Polymicrobial BSI	0	16 (7.0)	.001
BSI source			
Endovascular	20 (15.5)	64 (27.9)	.008
Infective endocarditis	20 (15.5)	54 (23.6)	.070
Other endovascular	0	11 (4.8)	.009
Intra-abdominal	0	1 (0.4)	1.000
Lower respiratory tract	6 (4.7)	72 (31.4)	<.001
Bone/joint	30 (23.3)	23 (10.0)	.001
Invasive prosthetic device	7 (5.4)	13 (5.7)	.921
Skin/soft tissue	39 (30.2)	37 (16.2)	.002
CNS abscess	5 (3.9)	4 (1.7)	.293
Intravenous catheter	24 (18.6)	49 (21.4)	.529
Urinary	3 (2.3)	6 (2.6)	1.000
Unknown	10 (7.8)	23 (10.0)	.472
Treatment data			
Infectious diseases consult	103 (79.8)	197 (86.0)	.128
Source control pursued	57 (44.2)	80 (34.9)	.084
Vancomycin TDM target			.015
Trough concentration 15–20 mg/L	115 (89.1)	181 (79.0)	
AUC 400–600 mg*h/L	14 (10.9)	48 (21.0)	
Cefepime dose, mg			—
1000	—	120 (52.4)	
2000		109 (47.6)	
Cefepime dose interval			
Every 6 h		2 (0.9)	
Every 8 h		95 (41.5)	
Every 12 h	—	52 (22.7)	—
Every 24 h		62 (27.1)	
Post-hemodialysis		18 (7.9)	
Inpatient vancomycin duration, d	6 (4–10)	5 (4–9)	.071
Inpatient cefepime duration (n = 229), d	—	3 (2–4)	—
Switched to daptomycin	36 (27.9)	70 (30.6)	.597
Switched to ceftaroline	8 (6.2)	25 (10.9)	.139
Switched to linezolid	8 (6.2)	13 (5.7)	.839
Switched to alternative anti-MRSA therapy before day 5	8 (6.2)	22 (9.6)	.264
Total duration inpatient antibiotics, d	9 (5–18)	9 (6–13.5)	.335
Outcomes			
Microbiologic failure	49 (38.0)	58 (25.3)	.012
BSI duration ≥7 d	40 (31.0)	43 (18.8)	.008
60-d MRSA BSI recurrence	15 (11.6)	19 (8.3)	.302
30-d mortality	10 (7.8)	47 (20.5)	.002
BSI duration, d	4 (3–7)	3 (2–5.5)	.003
LOS post–BSI onset, d	13 (8–21)	11 (7–17)	.064
Vancomycin-associated nephrotoxicity^h^	7 (5.4)	12 (5.2)	.940
Neurotoxicity attributed to antibiotic(s)^i^	0	1 (0.4)	1.000
*Clostridium difficile* infection^j^	2 (1.6)	8 (3.5)	.341

Abbreviations: APACHE II, Acute Physiology and Chronic Health Evaluation II; AUC, area under the concentration time curve; BSI, bloodstream infection; CNS, central nervous system; IV, intravenous; LOS, length of stay; MIC, minimum inhibitory concentration; MRSA, methicillin-resistant *Staphylococcus aureus*; TDM, therapeutic drug monitoring.

^a^Data presented as number (percentage) or median (interquartile range).

^b^Mild liver disease defined as chronic hepatitis without cirrhosis.

^c^Severe liver disease defined as portal hypertension or cirrhosis.

^d^Moderate/severe renal disease defined as chronic kidney disease stage 3 or greater or receiving chronic dialysis.

^e^Calculated using the Cockroft-Gault formula using actual body weight for body mass index <30 and adjusted body weight for body mass index >30.

^f^At time of index MRSA blood culture.

^g^Automated susceptibility testing performed by Microscan or Phoenix.

^h^Vancomycin-associated nephrotoxicity defined as a serum creatinine increase of 0.5 mg/L and 50% from baseline on 2 consecutive measurements from initial vancomycin dose to 72 hours after the last dose.

^i^Neurotoxicity defined as seizure, encephalopathy, or altered mental status specifically attributed to vancomycin and/or cefepime by treating physician(s).

^j^ *Clostridium difficile* infection defined as signs/symptoms along with positive laboratory test at least 48 hours after initiation of study antibiotics.

The results of logistic regression analysis for independent predictors of microbiologic failure are displayed in Table 2. Accounting for endovascular BSI source, African American race, and unknown BSI source, vancomycin + cefepime therapy was independently associated with less frequent microbiologic failure (adjusted odds ratio [aOR], 0.488; 95% confidence interval [CI], 0.271–0.741). Results were consistent in post hoc analysis in the cohort of 258 patients (129 vancomycin, 129 vancomycin + cefepime) who were matched on endovascular BSI source (Supplementary Table 2). Patients receiving vancomycin + cefepime were significantly less likely to experience microbiologic failure (aOR, 0.517; 95% CI, 0.298–0.899).

**Table 2.** Multivariable Logistic Regression for Factors Independently Associated With Microbiologic Failure

Variable	OR (95% CI)	Adjusted OR (95% CI)
Vancomycin + cefepime	0.554 (0.348–0.881)	0.488 (0.271–0.741)
Endovascular source	3.215 (1.930–5.356)	3.321 (1.925–5.730)
African American	2.542 (1.306–4.948)	2.121 (1.064–4.228)
Unknown source	0.297 (0.102–0.868)	0.360 (0.118–1.102)
Invasive prosthetic device source	2.004 (0.805–4.986)	—
Intravenous drug use	1.943 (1.072–3.523)	—
Bone/joint source	1.519 (0.826–2.791)	—
Chronic hemodialysis	1.484 (0.896–2.457)	—
Acute kidney injury	1.404 (0.875–2.253)	—
Lower respiratory tract source	0.487 (0.263–0.901)	—

Hosmer-Lemeshow goodness-of-fit test *P* = .835; variance inflation factor 1–5 for all variables included at model entry.

Abbreviations: CI, confidence interval; OR, odds ratio.

Because the difference in microbiologic failure was primarily due to BSI duration ≥7 days, logistic regression analysis was also performed for this outcome (Supplementary Table 3). Accounting for endovascular source, vancomycin + cefepime therapy was associated with reduced incidence of BSI duration ≥7 days (aOR, 0.354; 95% CI, 0.202–0.621). The results of logistic regression analysis for independent predictors of mortality are displayed in Table 3. Accounting for lower respiratory tract BSI source, endocarditis source, APACHE II score, age, and ID consult, vancomycin + cefepime therapy was not associated with 30-day mortality (aOR, 0.952; 95% CI, 0.435–2.425).

**Table 3.** Multivariable Logistic Regression for Factors Independently Associated With 30-Day Mortality

Variable	OR (95% CI)	Adjusted OR (95% CI)
Vancomycin + cefepime	3.073 (1.495–6.317)	1.023 (0.435–2.425)
Lower respiratory tract source	4.412 (2.419–8.046)	3.808 (1.799–8.057)
APACHE II	1.109 (1.074–1.146)	1.081 (1.043–1.121)
Age	1.054 (1.032–1.077)	1.041 (1.016–1.066)
Endocarditis	1.635 (0.858–3.116)	2.767 (1.216–6.073)
ID consult	0.467 (0.238–0.914)	0.502 (0.222–1.133)
Charlson comorbidity index	1.146 (1.026–1.280)	
Source control	0.332 (0.165–0.667)	

Hosmer-Lemeshow goodness-of-fit test *P* = .252; variance inflation factor 1–5 for all variables included at model entry.

Abbreviations: APACHE II, Acute Physiology and Chronic Health Evaluation II; CI, confidence interval; ID, Infectious Diseases; OR, odds ratio.

The results of proportional hazards regression models for BSI clearance are shown in Table 4. Accounting for endovascular source, patients receiving vancomycin + cefepime were more likely to experience BSI clearance (HR, 1.408; 95% CI, 1.125–1.762). Similar results were observed using a subdistribution hazard model (HR, 1.264; 95% CI, 1.040–1.536).

**Table 4.** Proportional Hazards Models for Bloodstream Infection Clearance

Variable	Hazard Ratio (95% CI)	*P* Value
Cause-specific hazard model^a^		
Vancomycin + cefepime	1.408 (1.125–1.762)	.003
Endovascular source	0.542 (0.418–0.703)	<.001
Subdistribution hazard model^b^		
Vancomycin + cefepime	1.264 (1.040–1.536)	.019
Endovascular source	0.569 (0.457–0.708)	<.001

Abbreviation: CI, confidence interval.

^a^African American race, unknown source, invasive prosthetic device source, bone/joint source, lower respiratory tract source, intravenous drug use, chronic hemodialysis, and acute kidney injury were all included at model entry but did not meet criteria for retention.

^a^Based on the results of the cause-specific model, only vancomycin + cefepime and endovascular were included in the model.

## DISCUSSION

This study sought to examine the impact of concomitant cefepime on the outcomes of patients with MRSA BSIs initially treated with vancomycin. The results suggest that concomitant cefepime improved BSI clearance, reducing the number of patients experiencing BSI durations ≥7 days. Although a larger proportion of patients in the vancomycin + cefepime group experienced 30-day mortality, this difference did not persist after accounting for clinical factors influencing mortality risk, such as age, infection source, and severity of illness. Care was also taken to ensure that the reduced BSI duration in the vancomycin + cefepime group was not due to the differential mortality in the combination group through cause-specific and subdistribution hazard models designed to account for this competing risk.

These data contribute to a growing body of clinical evidence that receipt of concomitant vancomycin and β-lactam antibiotics in patients with MRSA BSI improves BSI clearance and may improve microbiologic outcome. This includes a pilot randomized controlled trial of vancomycin + flucloxacillin and multiple small observational cohort studies of vancomycin in combination with various β-lactams [18–20]. Like the currently published observational studies, the β-lactam combination therapy in this present study was not given with the intent of providing synergy against MRSA but to provide broad-spectrum coverage. Although this diminishes the ability to measure the potential impact of a purposeful vancomycin + β-lactam combination therapy approach, it provides evidence that the in vitro synergy between vancomycin and β-lactams translates into patients. The present study advances knowledge of vancomycin + β-lactam combination therapy in 2 important ways. First, it focuses on cefepime, whereas previous studies included a heterogenous group of β-lactams, making it difficult to surmise which β-lactams confer benefit. Second, it accounts for the differential mortality risk typically seen in combination patients, which could otherwise be posited as a potential explanation for the observed shortened BSI duration with combination therapy (i.e., combination patients may have had shorter BSI durations because they were more likely to die before clearance of BSI could occur or be confirmed).

Although vancomycin + cefepime combination therapy improved MRSA BSI clearance, this does not indicate that cefepime should be the β-lactam of choice for directed MRSA BSI combination regimens. In vitro data coupled with pilot data of flucloxacillin suggest that antistaphylococcal penicillins and cefazolin can likely be used with vancomycin for directed MRSA BSI synergy [14–16, 18]. This would limit the potential unnecessary collateral damage that may come with the expanded gram-negative spectrum of cefepime. However, if a vancomycin + β-lactam approach is to be implemented, cefepime may be an ideal candidate for empiric therapy, provided that the institution’s gram-negative antibiogram permits. Because it is usually 24 hours before staphylococci are identified and often an additional 24–48 hours to detect methicillin resistance, MRSA-directed combination therapy may be delayed. Considering the importance of the initial 48 hours of therapy in *Staphylococcus aureus* bloodstream infection mortality, it would be ideal to provide empiric synergy [29–31]. In situations where empiric vancomycin + cefepime is already warranted, cefepime would satisfy the synergistic β-lactam role in patients ultimately found to have MRSA BSI. Upon isolation of *Staphylococcus aureus* or MRSA, cefepime could be switched to antistaphylococcal penicillin or cefazolin. If ongoing gram-negative coverage were required, synergy could be provided by continuing cefepime without the need to add a second β-lactam.

There are a number of considerations to bear in mind when interpreting these findings. Most importantly, the intention of the concomitant cefepime therapy was to provide either empiric or directed coverage of gram-negative organisms rather than synergy against MRSA. As such, it is unclear what the impact of purposeful vancomycin + β-lactam therapy would have been. This fact also resulted in considerable selection bias between the treatment groups, with vancomycin + cefepime patients having a substantially higher baseline mortality risk. Although various statistical techniques were used to account for this, the extraordinarily disproportionate mortality risk between treatment groups may have precluded our ability to observe a potential mortality benefit with combination therapy. Although no studies to date have demonstrated improvement in hard outcomes with vancomycin + β-lactam combination therapy for MRSA BSI, the known link between prolonged BSI and mortality make it reasonable to hypothesize that there is some benefit to a combination approach [32, 33]. Whether the effect size of this benefit is large enough to be feasibly measured in a clinical trial or is clinically meaningful is unknown. We did not observe increased adverse drug reactions in the combination group to indicate that the risk of combination may outweigh the potential benefit. However, it should be noted that this analysis was not specifically designed or powered to fully evaluate safety.

It is also important to note that the study population was derived from a single health system in Southeastern Michigan, which may limit the external generalizability of the findings. In particular, the MRSA strain epidemiology and practice pattern may differ from other sites, and it is unclear how this could have influenced the findings. The automated susceptibility testing MIC results from the hospital clinical microbiology laboratory did not indicate a difference in vancomycin susceptibility between the treatment groups. However, the Microscan platform used for the majority of the study period is known to consistently overcall vancomycin MIC relative to nonautomated broth microdiluton [34, 35]. This likely explains the large proportion of isolates with a vancomycin MIC of 2 mg/L in this study; this limited our ability to fully control for phenotype. Finally, because only a small proportion of the patients were monitored by vancomycin AUC, we were unable to control for vancomycin exposure [36]. Given the fact that AUC-guided dosing was more common in the vancomycin + cefepime group and is associated with reduced vancomycin dose and exposure, it is implausible that AUC-guided dosing would explain the improved BSI clearance observed in the combination patients [28, 37].

In conclusion, cefepime given concomitantly during the first 72 hours of vancomycin therapy was associated with improved MRSA BSI clearance. Although the cefepime was not given with the intention of providing synergy against MRSA, these data lend further support to the notion that vancomycin + β-lactam therapy may be a potential avenue to improve treatment outcomes of MRSA BSI. Although further study evaluating the impact of purposeful vancomycin + β-lactam therapy for MRSA BSI is needed before widespread clinical implementation, this study suggests that cefepime can be used as the β-lactam component of synergistic vancomycin + β-lactam regimens when empiric or directed gram-negative coverage is desired.

## Supplementary Data

Supplementary materials are available at *Open Forum Infectious Diseases* online. Consisting of data provided by the authors to benefit the reader, the posted materials are not copyedited and are the sole responsibility of the authors, so questions or comments should be addressed to the corresponding author.

ofz079_Suppl_Supplementary_Data
