# Corrigendum to: The Impact of Concomitant Empiric Cefepime on Patient Outcomes of Methicillin-Resistant *Staphylococcus aureus* Bloodstream Infections Treated With Vancomycin

**DOI:** 10.1093/ofid/ofaa059

**Published:** 2020-06-15

**Authors:** Evan J Zasowski, Trang D Trinh, Safana M Atwan, Marina Merzlyakova, Abdalhamid M Langf, Sahil Bhatia, Michael J Rybak

**Affiliations:** 1 Anti-Infective Research Laboratory, Department of Pharmacy Practice, Eugene Applebaum College of Pharmacy and Health Sciences, Wayne State University, Detroit, Michigan; 2 Department of Pharmacy Practice and Translational Research, University of Houston College of Pharmacy, Houston, Texas; 3 Department of Clinical Sciences, Touro University California College of Pharmacy, Vallejo, California; 4 Department of Clinical Pharmacy, UCSF School of Pharmacy, San Francisco, California; 5 Division of Infectious Diseases, Department of Medicine, School of Medicine, Wayne State University, Detroit, Michigan; 6 Department of Pharmacy Services, Detroit Medical Center, Detroit, Michigan

Open Forum Infectious Diseases, Volume 6, Issue 7, July 2019, ofz077, https://doi.org/10.1093/ofid/ofz077

In “The Impact of Concomitant Empiric Cefepime on Patient Outcomes of Methicillin-Resistant *Staphylococcus aureus* Bloodstream Infections Treated with Vancomycin”, Open Forum Infectious Diseases, Volume 6, Issue 7, July 2019, ofz077, eCollection 2019 July, in Table 3, the adjusted odds ratio (95% CI) for vancomycin + cefepime should have read 1.023 (0.435–2.425) and the adjusted odds ratio (95% CI) for endocarditis should have read 2.767 (1.216–6.073). The correct version of the table is displayed below. The noted changes do not alter the findings or conclusions. The authors regrets this mistake.

**Table 3.** Multivariable Logistic Regression for Factors Independently Associated With 30-Day Mortality

Variable	OR (95% CI)	Adjusted OR (95% CI)
Vancomycin + cefepime	3.073 (1.495–6.317)	1.023 (0.435–2.425)
Lower respiratory tract source	4.412 (2.419–8.046)	3.808 (1.799–8.057)
APACHE II	1.109 (1.074–1.146)	1.081 (1.043–1.121)
Age	1.054 (1.032–1.077)	1.041 (1.016–1.066)
Endocarditis	1.635 (0.858–3.116)	2.767 (1.216–6.073)
ID consult	0.467 (0.238–0.914)	0.502 (0.222–1.133)
Charlson comorbidity index	1.146 (1.026–1.280)	
Source control	0.332 (0.165–0.667)	

Hosmer-Lemeshow goodness-of-fit test *P* = .252; variance inflation factor 1–5 for all variables included at model entry.

Abbreviations: APACHE II, Acute Physiology and Chronic Health Evaluation II; CI, confidence interval; ID, Infectious Diseases; OR, odds ratio.

