# Supplementary material for: The Impact of Concomitant Empiric Cefepime on Patient Outcomes of Methicillin-Resistant Staphylococcus aureus Bloodstream Infections Treated With Vancomycin
Source: Open Forum Infect Dis. 2019 Apr 3;6(4):ofz079. doi: 10.1093/ofid/ofz079 (PMC6446134; doi:10.1093/ofid/ofz079)
Supplement: ofz079_Suppl_Supplementary_Data [file ofz079_suppl_supplementary_data.docx]

**SUPPLEMENTARY DATA**

**Supplementary Table 1**. Univariate description of demographic, clinical characteristics, and outcomes of included patients

| **Covariate** | **Observed Data^a^**  **(*n* = 358)** |
| --- | --- |
| **Demographics** |  |
| Age (years) | 60 (52-69) |
| Male | 232 (64.8) |
| Race  African American  Caucasian  Hispanic  Other/unknown | 285 (79.6)  65 (18.2)  5 (1.4)  3 (0.8) |
| **Comorbidities & Past Medical History** |  |
| Myocardial Infarction | 24 (6.7) |
| Heart Failure | 84 (23.5) |
| Peripheral vascular disease | 70 (19.6) |
| Cerebrovascular disease | 63 (17.6) |
| Dementia | 44 (12.3) |
| Chronic pulmonary disease  Chronic obstructive pulmonary disease  Asthma | 91 (25.4)  80 (22.3)  17 (4.7) |
| Connective tissue disease | 43 (12.0) |
| Peptic ulcer disease | 7 (2.0) |
| Liver disease  Mild^b^  Moderate/severe^c^ | 56 (15.6)  50 (14.0)  6 (1.7) |
| Diabetes  With end-organ damage | 136 (38.0)  93 (26.0) |
| Hemiplegia | 8 (2.2) |
| Moderate/severe renal disease^d^  Chronic hemodialysis | 127 (35.5)  91 (25.4) |
| Solid tumor without metastasis | 7 (2.0) |
| Leukemia | 0 |
| Lymphoma | 0 |
| Metastatic solid tumor | 13 (3.6) |
| Human immunodeficiency virus | 12 (3.4) |
| Acquired immune deficiency syndrome | 5 (1.4) |
| Charlson Comorbidity Index | 3 (1-5) |
| Intravenous drug use | 54 (15.1) |
| Prior hospitalization (90 days) | 145 (40.5) |
| Prior IV vancomycin (90 days) | 82 (22.9) |
| Prior MRSA infection (1 year) | 54 (15.1) |
| **Clinical Data** |  |
| Admitted from  Home  Nursing home/SNF/LTAC  Transferred from another hospital | 244 (68.2)  88 (24.6)  26 (7.3) |
| Weight (kg) | 76.2 (66.2-90.5) |
| Creatinine clearance^e,f^ (mL/min) | 64.3 (40.9) |
| Acute kidney injury^f^ | 118 (33.0) |
| APACHE II score^f^ | 18 (11-24) |
| Neutropenia^f^ | 1 (0.3) |
| **Infection Data** |  |
| Vancomycin MIC^g^  2 mg/L  1 mg/L  ≤ 0.5 mg/L | 146 (40.8)  209 (58.4)  3 (0.8) |
| Polymicrobial BSI | 16 (4.5) |
| Endovascular  Infective endocarditis  Other endovascular | 84 (23.5)  74 (20.7)  11 (3.1) |
| Intra-abdominal | 1 (0.3) |
| Lower respiratory tract | 77 (21.5) |
| Bone/joint | 53 (14.8) |
| Invasive prosthetic device | 20 (5.6) |
| Skin/soft tissue | 76 (21.2) |
| CNS abscess | 9 (2.5) |
| Intravenous catheter | 73 (20.4) |
| Urinary | 9 (2.5) |
| Unknown | 32 (8.9) |
| **Treatment Data** |  |
| Infectious Diseases Consult | 300 (83.8) |
| Source control pursued | 137 (38.3) |
| Vancomycin + cefepime | 229 (64.0) |
| Vancomycin TDM target  Trough concentration 15-20 mg/L  AUC 400 to 600 mg*h/L | 420 (79.5)  108 (20.5) |
| Cefepime dose – (*n* = 229)  1000 mg  2000 mg | 120 (52.4)  109 (47.6) |
| Cefepime dose interval – (*n* = 229)  Every 6 hours  Every 8 hours  Every 12 hours  Every 24 hours  Post-hemodialysis | 2 (0.9)  95 (41.5)  52 (22.7)  62 (27.1)  18 (7.9) |
| Inpatient vancomycin duration (days) | 5 (4-9) |
| Inpatient cefepime duration (days) – (*n* = 229) | 3 (2-4) |
| Switched to daptomycin | 106 (29.6) |
| Switched to ceftaroline | 33 (9.2) |
| Switched to linezolid | 21 (5.9) |
| Switched to alternative anti-MRSA therapy before day 5 | 30 (8.4) |
| Total duration inpatient antibiotics (days) | 9 (6 – 14) |
| **Outcomes** |  |
| Microbiologic failure  BSI duration ≥ 7 days  60-day MRSA BSI recurrence | 107 (29.9)  83 (23.2)  34 (9.5) |
| 30-day mortality | 57 (15.9) |
| BSI duration (days) | 4 (2-6) |
| LOS post-BSI onset (days) | 11 (7-18.3) |
| Vancomycin-associated nephrotoxicity^h^ | 19 (5.3) |
| Neurotoxicity attributed to antibiotic(s)^i^ | 1 (0.3) |
| Clostridium difficile infection^j^ | 10 (2.8) |

Abbreviations: IV, intravenous; MRSA, methicillin-resistant *Staphylococcus aureus*; APACHE, acute physiology and chronic health evaluation; MIC, minimum inhibitory concentration, a BSI, bloodstream infection; CNS, central nervous system; TDM, therapeutic drug monitoring; AUC, area under the concentration-time curve; LOS, length of stay

^a^ Data presented as number (percentage) or median (interquartile range)

^b^ Mild liver disease defined as chronic hepatitis without cirrhosis

^c^ Severe liver disease defined as portal hypertension or cirrhosis

^d^ Moderate/severe renal disease defined as chronic kidney disease stage 3 or greater or receiving chronic dialysis

^e^ Calculated using cockroft-gault formula using actual body weight for body mass index < 30 and adjusted body weight for body mass index > 30

^f^ At time of index MRSA blood culture

^g^ Automated susceptibility testing performed by Microscan or Phoenix

^h^ Vancomycin-associated nephrotoxicity defined as a serum creatinine increase of 0.5 mg/L and 50% from baseline on two consecutive measurement from initial vancomycin dose to 72 hours after the last dose

^i^ Neurotoxicity defined as seizure, encephalopathy, or altered mental status specifically attributed to vancomycin and/or cefepime by treating physician(s)

^j^ Clostridium difficile infection defined as signs/symptoms along with positive laboratory test at least 48 hours after initiation of study antibiotics

**Supplementary Table 2.** Conditional multivariable logistic regression for factors independently associated with microbiologic failure in cohort of patients matched on endovascular bloodstream infection source

| **Variable** | **OR (95% CI)** | **Adjusted OR (95% CI)** |
| --- | --- | --- |
| Vancomycin + cefepime | 0.516 (0.302 – 0.885) | 0.517 (0.298 – 0.899) |
| Endovascular source | 2.633 (1.324 – 5.236) | 2.608 (1.293 – 5.259) |
| Unknown source | 0.263 (0.076 – 0.902) | 0.292 (0.083 – 1.027) |
| African American | 2.287 (1.051 – 4.977) | - |
| Intravenous drug use | 1.725 (0.800 – 3.719) | - |
| Invasive prosthetic device source | 2.749 (0.893 – 8.461) | - |
| Bone/joint source | 1.685 (0.835 – 3.397) | - |

Abbreviations: OR, odds ratio, CI, confidence interval

*n* = 258; 129 vancomycin; 129 vancomycin + cefepime

Hosmer-Lemeshow goodness-of-fit test *P* = 0.961; variance inflation factor 1-5 for all variables included at model entry

**Supplementary Table 3.** Multivariable logistic regression for factors independently associated with BSI duration ≥ 7 days

| **Variable** | **OR (95% CI)** | **Adjusted OR (95% CI)** |
| --- | --- | --- |
| Vancomycin + cefepime | 0.514 (0.312 – 0.847) | 0.354 (0.202 – 0.621) |
| Endovascular source | 4.530 (2.649 – 7.747) | 5.852 (3.273 – 10.462) |
| African American | 2.169 (1.058 – 4.449) | - |
| Intravenous drug use | 2.036 (1.092 – 3.795) | - |
| Invasive prosthetic device source | 1.856 (0.715 – 4.817) | - |
| Bone/joint source | 1.710 (0.904 – 3.236) | - |
| Unknown source | 0.306 (0.091 – 1.030) | - |
| Lower respiratory tract source | 0.417 (0.204 – 0.853) | - |

Abbreviations: OR, odds ratio, CI, confidence interval

Hosmer-Lemeshow goodness-of-fit test *P* = 0.772; variance inflation factor 1-5 for all variables included at model entry
